# Prediction of arterial pressure increase after fluid challenge

**DOI:** 10.1186/1471-2253-12-3

**Published:** 2012-03-05

**Authors:** Giuseppe Natalini, Antonio Rosano, Carmine Rocco Militano, Antonella Di Maio, Pierluigi Ferretti, Michele Bertelli, Federica de Giuli, Achille Bernardini

**Affiliations:** 1 Intensive Care Unit, Poliambulanza Hospital Foundation, Brescia, Italy; 2 Cardiology Division, Salvatore Maugeri Foundation, Lumezzane, Italy

## Abstract

**Background:**

Mean arterial pressure above 65 mmHg is recommended for critically ill hypotensive patients whereas they do not benefit from supranormal cardiac output values. In this study we investigated if the increase of mean arterial pressure after volume expansion could be predicted by cardiovascular and renal variables. This is a relevant topic because unnecessary positive fluid balance increases mortality, organ dysfunction and Intensive Care Unit length of stay.

**Methods:**

Thirty-six hypotensive patients (mean arterial pressure < 65 mmH) received a fluid challenge with hydroxyethyl starch. Patients were excluded if they had active bleeding and/or required changes in vasoactive agents infusion rate in the previous 30 minutes. Responders were defined by the increase of mean arterial pressure value to over 65 mmHg or by more than 20% with respect to the value recorded before fluid challenge. Measurements were performed before and at one hour after the end of fluid challenge.

**Results:**

Twenty-two patients (61%) increased arterial pressure after volume expansion. Baseline heart rate, arterial pressure, central venous pressure, central venous saturation, central venous to arterial PCO_2 _difference, lactate, urinary output, fractional excretion of sodium and urinary sodium/potassium ratio were similar between responder and non-responder. Only 7 out of 36 patients had valuable dynamic indices and then we excluded them from analysis. When the variables were tested as predictors of responders, they showed values of areas under the ROC curve ranging between 0.502 and 0.604. Logistic regression did not reveal any association between variables and responder definition.

**Conclusions:**

Fluid challenge did not improve arterial pressure in about one third of hypotensive critically ill patients. Cardiovascular and renal variables did not enable us to predict the individual response to volume administration.

**Trial registration:**

ClinicalTrials.gov: NCT00721604.

## Background

Fluid administration is the first recommended approach to increase arterial blood pressure in critically ill hypotensive patients [1-3]. If early aggressive fluid resuscitation is useful at the beginning of care in injured and septic patients [3,4], there is evidence that unnecessary fluid administration and positive fluid balance increase mortality, organ dysfunction and Intensive Care Unit length of stay when the whole period of care is considered [5-8]. To avoid fluid overload it is important to identify beforehand the patients for whom volume expansion would increase arterial pressure and those patients who do not benefit from fluid administration.

Previous studies have investigated the possibility of predicting fluid responsiveness through assessing increases in cardiac output after fluid challenge. Dynamic indices, in particular pulse pressure variation, assessed fluid responsiveness better than static indices, such as central venous pressure or pulmonary capillary wedge pressure [9-12]. Fluid responsiveness does not help to predict if patients actually increase arterial pressure after volume expansion but it identifies patients who increase their cardiac output. Moreover it should be considered that fluid responsiveness was mainly studied in critically ill patients with normal-to-high cardiac output [9-12] and any further increase in cardiac output does not improve prognosis in such patients [13,14].

In this study we investigated if the increase of arterial pressure after volume expansion could be predicted by cardiovascular and renal variables in hypotensive critically ill patients.

## Methods

### Patients

The protocol was approved by the institutional ethical committee (Comitato Etico delle Istituzioni Ospedaliere Cattoliche) and written consent was obtained by the patients or their next of kin if the patients themselves were not competent. Written consent was not be required in cases where this would delay urgent fluid challenge.

We studied 36 consecutive patients admitted to the Intensive Care Unit of Poliambulanza Foundation Hospital. Patients were recruited if they met the following criteria: mean arterial pressure lower than 65 mmHg, age over 18 years and presence of both central venous and arterial catheters. Excluded were unstable patients and patients during the early resuscitation phase suffering from hypovolemic shock or septic shock/severe sepsis. Accordingly patients were excluded if they had active bleeding and/or required changes in vasoactive agents infusion rate in the previous 30 minutes. Additional exclusion criteria were: central venous pressure higher than 16 mmHg, pulmonary congestion or edema, impending risk of death, or plasma hemoglobin lower than 8 g.dl^-1^.

### Measurements and calculations

Electrocardiography, mean arterial pressure, central venous pressure, pulse oximetry, were continuously monitored (Datex-Engstrom CS/3 Critical Care Monitor, Datex-Engstrom Division, Instrumentarium Corp., Helsinki, Finland). Values of heart rate, mean arterial pressure and central venous pressure (sampled every ten seconds), and waveforms of arterial pressure, central venous pressure and pulse oximetry plethysmography (sampling rate 100 Hz) were recorded for three minutes immediately before and at one hour after the end of fluid challenge and then converted to ASCII files (Datex-Ohmeda S/5 Collect, Datex-Ohmeda Division, Instrumentarium Corp., Helsinki, Finland). The definitive values of heart rate, mean arterial pressure and central venous pressure were the mean values of recorded data and were used for analysis. Data below the 10th centile or above the 90th centile were not included in mean calculation. Pulse pressure variation and pulse plethysmographic variation were calculated only in patients without both arrhythmias and spontaneous respiratory activity as evaluated by airway and flow waveforms. Calculations were performed as previously described [9,10,15].

Samples of arterial blood, central venous blood and urine were simultaneously collected just before and at one hour after the end of the fluid challenge. Central venous saturation, central venous to arterial CO_2 _partial pressure difference (Δv-aPCO_2_), arterial blood lactate, plasmatic and urinary creatinine, plasmatic and urinary sodium, urinary potassium and last hour urinary output were measured. Fractional excretion of sodium (FENa) was calculated as: [16].

### Protocol

A maximum of two consecutive fluid challenges was planned. After baseline measurements had been made, patients received a first fluid challenge that was immediately followed by a second fluid challenge, provided that central venous pressure was again lower than 16 mmHg and mean arterial pressure did not exceed 75 mmHg. Both fluid challenges were carried out with 7 ml.kg^-1 ^of 6% hydroxyethyl starch (Voluven, Fresenius Kabi Italia S.r.l., Isola della Scala, Italy) over 30 minutes. Hydroxyethyl starch infusion was stopped if one of the following conditions lasted more than 3 consecutive minutes: central venous pressure increased of more than 20% with respect to the basal value and with a value greater than 16 mmHg; mean arterial pressure greater than 75 mmHg; decrease of pulse oximetry saturation greater than 5%.

The goal of the fluid challenge was to restore mean arterial pressure value to over 65 mmHg or to increase it by more than 20% with respect to the value recorded before fluid challenge. The outcome was evaluated at one hour after the end of hydroxyethyl starch infusion. These patients were defined as responders to fluid challenge.

Throughout the protocol, the ventilator setting and vasoactive drug infusion were not changed. Patients who needed to increase vasoactive drug infusion before the end of the protocol were considered as non-responders.

### Study outcome

The main study outcome was to evaluate the accuracy of baseline physiological variables to identify responders to fluid challenge. The planned variables to test were central venous pressure, Δv-aPCO_2_, arterial lactate, FENa, urinary sodium/potassium ratio, pulse pressure variation, plethysmographic pulse variation.

### Statistics

Data are shown as mean ± sd, median (1-3 quartiles) or count (percentage) as appropriate. Differences in frequency were analyzed by the Fisher exact test. Values obtained before and after fluid challenge were compared by a paired *t *test or paired Wilcoxon test. Differences between responders and non-responders were evaluated by a *t *test or Wilcoxon test. Diagnostic performance was firstly evaluated by area under the ROC curve. Accuracy, positive and negative predictive value, sensitivity and specificity were calculated for variables which had an area of greater than 0.8 under the ROC curve. Comparisons between areas under ROC curve were performed only if their values were greater than 0.8. In order to identify variables associated with responders status, we performed univariate analysis and variables with p value lower than 0.1 were included as covariates in multiple logistic regression to estimate adjusted OR with their 95% CI. Statistical analyses were performed using R statistical software, version 2.14.0, with the package pROC (R Foundation for Statistical Computing, Vienna, Austria, http://www.R-project.org).

## Results

**Figure 1:**
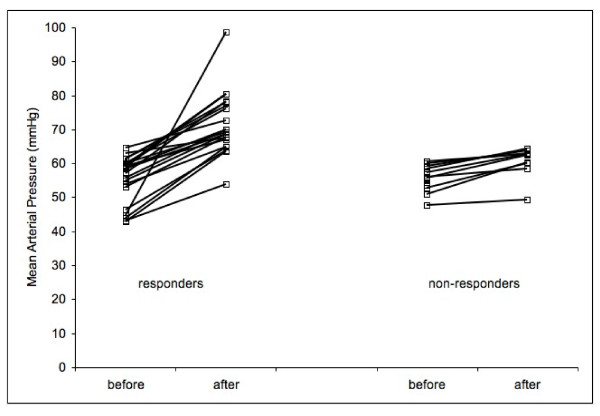
**Individual changes in mean arterial pressure before and after fluid challenge**.

Patient characteristics are shown in Table 1. All patients had mean arterial pressure lower than 65 mmHg before fluid challenge as set out in the inclusion criteria. All patients completed the first fluid challenge but seven out of 36 patients (19%) met the criteria to stop the second fluid challenge. Four patients required the commencement of vasoactive drug agent infusion or an increased infusion rate immediately after the end of fluid challenge. Individual changes in arterial pressure are shown in Figure 1.

**Table 1:** Patients characteristics

Age (years)	66 ± 18
Bodi Mass Index	25 ± 6
Predicted mortality by SAPS 2 (%)	51 (28-77)
Actual hospital mortality [n (%)]	13 (36)
Norepinephrine infusion rate (mcg.kg^-1^.min^-1^) (12 patients)	0.32 ± 0.13
Dobutamine infusion rate (mcg.kg^-1^.min^-1^) (2 patients)	4.5 ± 0.7
SOFA score on the study day	7 (5.8-10)
Spontaneous respiratory activity [n (%)]	27 (75)
Arrhythmias [n (%)]	6 (17)
Fluid challenge volume (l)	0.96 ± 0.24
Fluid challenge volume (ml.kg^-1^)	13 ± 2

ICU: Intensive Care Unit. SAPS: Simplified Acute Physiological Score; SOFA: Sequential Organ Failure Assessment. Data are shown as mean ± SD or median (interquartile range)

Before fluid challenges central venous pressure, central venous saturation and arterial lactate were available in all study patients (100%); Δv-aPCO_2 _and FENa were obtained in 35 out of 36 patients (97%); urinary sodium/potassium ratio was obtained for 31 patients (86%). Each of these variables was available more frequently than dynamic indices, which were usable in 7 out of 36 patients (19%) (p < 0.001). Dynamic indices were excluded from any further analysis because of their low availability.

Twenty-two out of 36 patients (61%) were responders to fluid challenge. The values of variables collected before and at one hour after fluid challenge are shown in Table 2. Fluid challenge increased mean arterial pressure and urinary output but does not central venous saturation. Central venous pressure was increased by fluid challenge, while the other cardiovascular and renal variables were not. The values of the variables obtained before fluid challenge were similar in responders and non-responders.

**Table 2:** Cardiovascular, urinary and metabolic variables

	All patients			Before fluid challenge		
	**Before fluid challenge**	**After fluid challenge**	**p**	**Non-responders**	**Responders**	**p**

Heart rate (beat.min^-1^)	81 ± 19	77 ± 17	0.08	78 ± 14	83 ± 21	0.43
Mean arterial pressure (mmHg)	57 (53-59)	67 (63-74)	< 0.001	56 (53-58)	59 (53-60)	0.45
Central venous pressure (mmHg)	8 ± 5	11 ± 6	< 0.001	5 (4-10)	9 (5-11)	0.31
Central venous saturation (%)	72 ± 10	73 ± 10	0.22	73 ± 8	71 ± 11	0.45
Arterial lactate (mMol.l^-1^)	1.3 (1-2.1)	1.3 (1-1.7)	0.022	1.5 (0.8-2.5)	1.3 (1-1.9)	1
Δv-aPCO_2_ (mmHg)	6 (3.5-7.5)	5(2.8-6.3)	0.58	6 ± 3	5 ± 4	0.42
Urinary output (ml.kg^-1^.h^-1^)	0.7 (0.4-1.2)	1.1 (0.6-1.6)	0.024	0.7 (0.4-1.2)	0.8 (0.4-1.1)	0.53
FENa (%)	0.3 (0.1-1.2)	0.4 (0.1-1.5)	1	0.3 (0.1-1.3)	0.4 (0.1-1.2)	0.77
Urinary Na/K ratio	0.9 (0.3-2.2)	0.8 (0.3-2.7)	0.48	0.6 (0.2-2.5)	1.1 (0.4-2.1)	0.54

Δv-aPCO_2_: central venous to arterial CO_2 _partial pressure difference; FENa: fractional excretion of sodium. Data are shown as mean ± SD or median (interquartile range). Δv-aPCO2 and FENa were evaluated in 35 patients, urinary Na/K ratio 31 patients

When the variables in Table 2 were tested as predictors of the effect of fluid challenge, they showed values of areas under the ROC curve ranging between 0.502 and 0.604. Finally, logistic regression did not reveal any association between variables and responder definition (Table 3).

**Table 3:** Predictive value and association between outcome (responder) and study variables

	Area under ROC curve (95% CI)	OR (95% CI)
Heart rate (beat.min^-1^)	0.564 (0.369-0.76)	1.01 (0.98-1.05)
Mean arterial pressure (mmHg)	0.578 (0.386-0.77)	1.01 (0.9-1.13)
Central venous pressure (mmHg)	0.604 (0.401-0.807)	1.09 (0.94-1.27)
Central venous saturation (%)	0.584 (0.391-0.777)	0.97 (0.91-1.04)
Arterial lactate (mMol.l^-1^)	0.502 (0.277-0.726)	0.81 (0.49-1.35)
Δv-aPCO_2 _(mmHg)	0.570 (0.365-0.775)	0.93 (0.77-1.12)
Urinary output (ml.kg^-1^.h^-1^)	0.571 (0.353-0.79)	1.25 (0.5-3.11)
FENa (%)	0.536 (0.301-0.771)	0.93 (0.63-1.4)
Urinary Na/K ratio	0.573 (0.341-0.804)	1.1 (0.79-1.53)

Δv-aPCO_2_: central venous to arterial CO_2 _partial pressure difference; FENa: fractional excretion of sodium. CI: confidence interval

## Discussion

This study aimed to identify predictors regarding the increase of arterial pressure after volume expansion. Fluid challenge was ineffective in about one third of patients and the cardiovascular and renal variables analyzed in this study did not allow any kind of prediction regarding the increase of arterial pressure.

### Study results

We assessed fluid responsiveness from a clinical instead of physiological point of view. Fluid administration is traditionally considered as effective if it increases cardiac output. This has a pathophysiological rationale and dynamic indices appear quite reliable predictors with this approach [11,12]. Nevertheless a frequent clinical reason for fluid administration is hypotension whereas cardiac output increase is not required in most of intensive care patients [13,14]. In our study dynamic indices were available for prediction only in 19% of the population. This finding was explained by the two main limitations of dynamic indices assessment, namely, the absence of both spontaneous respiratory activity and arrhythmias [9-11]. These requirements are frequently violated in critically ill patients. Arrhythmias have been reported in 15% of patients in Intensive Care Unit [17] and early resumption of some spontaneous respiratory activity is recommended to facilitate weaning from mechanical ventilation and to prevent ventilation-induced diaphragmatic dysfunction [18,19]. We did not consider the use of low tidal volume ventilation as exclusion criteria for use of dynamic indices. Low tidal volume ventilation is recommended during acute lung injury [3] but this ventilatory approach reduces the accuracy of fluid responsiveness prediction by dynamic indices [15,20,21]. If we considered even low tidal volume ventilation as exclusion criteria for dynamic indices evaluation, then dynamic indices would have been available in only 2 out of 36 patients (6%). Therefore dynamic indices could be a useful resource in non-critically ill patients, for example in perioperative settings. Indeed, spontaneous respiratory activity and low tidal volume ventilation are not frequently used during general anesthesia. Moreover, dynamic indices are a good predictor of fluid responsiveness evaluated by cardiac index increase, and supranormal values of cardiac index and oxygen delivery are also associated with better outcomes in high risk patients undergoing major surgery [22].

When we tried to predict the effect of fluid challenge, we obtained frustrating results. Before fluid challenge all variables were similar between responders and non-responders. Moreover, study variables displayed very low accuracy in identifying responders to fluid challenge, with their areas under ROC curves ranging from 0.502 to 0.604. Finally, even logistic regression did not identify any variables associated with responder status. These results further discourage the use of central venous pressure to drive fluid administration in hypotensive patients.

Most variables tested in this study depends mainly on pressure and flow. Therefore our results suggest that physiological variables depending on flow and pressure could not accurately predict arterial pressure changes after fluid administration. For this purpose it appears more appropriate an approach based on the arterial elastance evaluation as calculated by ratio between pulse pressure variation and stroke volume variation [23].

In previous studies fluid challenge increased cardiac output or stroke volume in 40-64% of patients [9,10,15,20,21] and similarly, in our study, fluid challenge worked in 61% of the patients. At least one in every three hypotensive patients does not show any improvement after fluid challenge. On the contrary, inappropriate fluid administration negatively impacts on several relevant outcomes [5-8]. Considering the impossibility of predicting the effect of volume expansion on arterial pressure and the high rate of non-responders, cardiac output monitoring or estimation could help to guide therapy in hypotensive patients whose arterial pressure does not increase after fluid challenge or who are at risk of fluid overload.

The results of this study strongly depended on fluid challenge technique used, the timing of outcome evaluation, study outcome, patient and variable selection.

### Fluid challenge

We chose to administer maximal fluid challenge to avoid any possibility of fluid underresuscitation. Each fluid challenge was scheduled to administer about 500 ml over 30 minutes for patients of 70 kg in weight [3,24]. Because some patients could require larger volumes [3], we decided to repeat the fluid challenge provided that there were no criteria for stopping fluid administration. The total amount of hydroxyethyl starch remained largely below the safety threshold dose even when both fluid challenges were carried out [25].

### Timing

Previous studies on fluid responsiveness have evaluated the outcome either at the end of volume infusion [9,15,20,21] or at 30 minutes after it [10]. We performed outcome measurements at 1 hour after the end of fluid challenge to avoid misclassification of transient effects as clinically relevant effects. At this time, the volume effect of hydroxyethyl starch was fully maintained [25]. Moreover timing of measurement of effect of volume expansion on cardiac output and arterial pressure could strongly influence results. In fact changes of cardiac output and arterial pressure after fluid challenge have different time course. In preload-dependent patients cardiac output can initially increase by a large extent despite minimal arterial pressure increase because of decrease in systemic vascular resistance. Then cardiac output returns to baseline value and arterial pressure increases for the combined effect of an increase of venous capacitance associated to disappearance of initial decrease in systemic vascular resistance. This dynamic process begins immediately after the fluid challenge and it is complete in few hours. In particular the evaluation of fluid responsiveness should be delayed at least 40 minutes after the fluid challenge to allow the first acute stabilization of cardiac output and arterial pressure [26].

### Patients

Fluid administration should follow different approaches in the first hours of resuscitation and after the stabilization of the patients [3,4,27]. If, in the first hours, more fluid is better than less [4], liberal fluid administration should be avoided in the following phases [5-8]. We aimed to study only stable patients after the start phase of fluid resuscitation. We avoided selecting patients with specific clinical diagnosis because they are often extremely heterogeneous from a pathophysiologic point of view. For example, patients with septic shock/severe sepsis are probably the most studied population of critically ill hypotensive patients. All these patients share an infection-related hypotension but they should be considered a heterogeneous population when they are enrolled in a physiological study. It is well recognized that they could have low or normal or high cardiac preload associated with low or normal or high cardiac output [3]. Therefore we chose to increase the external validity of the study both avoiding apparent homogeneity and including patients independently of cardiac output measurement, cardiac rhythms and modality of ventilation. This study should be considered as a pilot trial and the sample size was considered convenient for this purpose. Nevertheless these preliminary data have shown that predictors were much too weak for a successful prediction even in much more patients.

### Study outcome

The study goal was to improve arterial pressure not cardiac output because the indication for fluid challenge was hypotension. We defined as responder those patients whose mean arterial pressure reached values higher than 65 mmHg [1-3] or increased by more than 20%. In this manner we included among the responder group those patients who showed large arterial pressure increases but who did not reach the 65 mmHg threshold because they had started with very low basal arterial pressure values.

### Variables

We chose only variables that were reliable and available in most of the critically ill patients and were related to tissue perfusion or recommended by guidelines on fluid administration in hypotensive patients [3,12,15,16,28-30]. Measurement of cardiac output could give additional useful information. Nevertheless in daily practice this information is suitable only in selected patients: consequently the external validity of the study would be strongly reduced if patient enrollment would be limited to those with cardiac output monitoring. Moreover we analyzed the urinary sodium/potassium ratio because it is sometimes used in clinical practice despite a lack of evidence to support it. We cannot exclude the possibility that analyzing different variables could enable the reliable prediction of the effect of fluid challenge.

## Conclusion

Fluid challenge did not improve arterial pressure in about one third of hypotensive critically ill patients. Cardiovascular and renal variables did not enable us to predict the individual response to volume administration.

## Abbreviations

FENa: Fractional excretion of sodium; ROC: Receiver operating characteristic; Δv-aPCO_2: _Central venous to arterial CO_2 _partial pressure difference.

## Competing interests

The authors declare that they have no competing interests.

## Authors' contributions

GN conceived of the study, participated in its design, conducted the study, performed statistical analysis and wrote the manuscript. AR participated in the design of the study and conducted the study. CRM analyzed the data. ADM helped to conduct the study. PF helped to conduct the study. MB helped to conduct the study. FdG participated in the design of the study, helped to conduct the study and analyzed the data. AB participated in the design of the study. All authors read and approved the final manuscript.

## Pre-publication history

The pre-publication history for this paper can be accessed here:

http://www.biomedcentral.com/1471-2253/12/3/prepub
